# Nonsuicidal Self-Injury and Perfectionism: A Systematic Review

**DOI:** 10.3389/fpsyt.2021.691147

**Published:** 2021-07-07

**Authors:** Dora Gyori, Judit Balazs

**Affiliations:** ^1^Doctoral School of Psychology, Eotvos Lorand University, Budapest, Hungary; ^2^Institute of Psychology, Eotvos Lorand University, Budapest, Hungary; ^3^Department of Psychology, Bjørknes University College, Oslo, Norway

**Keywords:** NSSI, nonsuicidal self-injury, perfectionism, review, clinical sample, community sample

## Abstract

**Background:** Nonsuicidal self-injury (NSSI) and perfectionism mean a huge concern related to mental health and psychopathology. Recently, there has been a growing interest in research on the exploration of the association of perfectionism and NSSI, but till today there is no systematic review has been prepared in this topic.

**Aims:** Therefore, we performed a systematic literature review of published studies that investigated the association between NSSI and perfectionism.

**Methods:** The systematic search was made on PubMed, OVID Medline, PsychInfo, Scopus, and Web of Science. The search terms were (“nonsuicidal self-injury” OR “nonsuicidal self-injury” OR NSSI OR “self-injury” OR “self-injurious behavior” OR SIB OR “self-harm” OR “deliberate self-harm” OR DSH) AND (perfectionism). The inclusion criteria were as follows: written in English; reported empirical data; used validated self-report measures; investigated the association of nonsuicidal self-injury and perfectionism. There were no restrictions on participants regarding age, gender, race or ethnicity. Exclusion criteria: not written in English; was a review/meta-analysis; measured suicide behavior; measured self-injury irrespective of motivation or suicidal intent; was not about the association between nonsuicidal self-injury and perfectionism.

**Results:** After the screening process, 15 studies were included in our systematic review. The majority of studies (12) were published in the last 10 years. Nine (60%) recruited participants from community samples, four (26.7%) from clinical populations, and two (13.3%) both from community and clinical participants. Fourteen (93.3%) of the studies were cross-sectional studies, and one study contained a longitudinal investigation. The majority of studies included only or mainly female participants (62.3–87.2%) and two studies contained a balanced male-female ratio population. Fourteen (93.3%) studies from the 15 studies found a significant positive association between NSSI and perfectionism.

**Limitations:** The heterogeneity of used instruments makes it difficult to compare the results of involved studies. Only two studies investigated populations with balanced gender ratios. Only two studies examined both clinical and community populations. Clinical investigations enrolled mainly eating-disordered (ED) patients.

**Conclusions:** The results of the current systematic review highlight the role of perfectionism in NSSI engagement. This systematic review may help the development of effective prevention initiatives and treatment strategies.

## Introduction

Nonsuicidal self-injury (NSSI) is a growing clinical and mental health problem, especially for youth and young adults (1–3). It is characterized by intentional self-inflicted damage to body tissue without suicidal intent (such as cutting, burning, scraping skin, hitting, and biting oneself), which is not culturally sanctioned behavior (4, 5). Nonsuicidal self-injury disorder (NSSI-D) was introduced in the Diagnostic and Statistical Manual of Mental Disorders 5th Edition (DSM-5), under section III, “Conditions for Further Study (5).” According to the diagnostic criteria, self-injurious acts should be completed on five or more separate days in the past year. Expectations of people who engage in NSSI actions is that self-injury behavior provides (a) relief of a negative affective/cognitive state after the NSSI, or (b) resolution of interpersonal difficulties or (c) cause positive feelings (5).

NSSI engagement generally occurs in early adolescent years; the typical age of onset is 12–14 years (6). NSSI prevalence increases in early adolescence; the peak is around 15–17 years, so it is a serious mental health problem among youths (6, 7). In late adolescence, NSSI prevalence decreases (1). Prevalence in community adolescents was found to be 17–46.5% (2, 8–10) and in clinical samples of adolescents was 60–80% (11, 12). Among adult community samples, NSSI prevalence was 4–23% (2, 13, 14). A meta-analysis concluded that NSSI incidence is more common among women compared to men (15).

The most common forms of NSSI behavior include cutting, scratching, burning, head banging, self-hitting and biting (2, 4, 16). The majority of individuals who engaged in NSSI use more than one methods (17). NSSI behavior serves multiple psychological functions: affect regulation, self-punishment, interpersonal influence, anti-dissociation, anti-suicide, sensation seeking, interpersonal boundaries (4), and affect regulation and self-punishment are the most common functions (18). Previous study concluded that the majority of individuals use multiple functions (12, 19).

In the literature there is great heterogeneity of definitions of self-injurious behaviors (1, 20), and there have been described different terms regardless of their suicidal intent (1, 20). In contrast, the NSSI definition contains behaviors without suicidal intent (5).

Perfectionism is commonly defined as “the setting of excessively high standards of performance [21, p. 450],” which is accompanied by overly self-critical evaluations (21). The literature emphasizes the multidimensional nature of this construct with personal and social dimensions (21, 22). Multidimensional measurements have been developed.

One of the two widely used measures of perfectionism was developed by Frost et al. (21). The Frost Multidimensional Perfectionism Scale (FMPS) proposed six facets of perfectionism: concern over mistakes (CM, reacting negatively in case of mistakes, interpret mistakes as failure), doubts about actions (DA, doubting related to the ability of one's performance), parental criticism (PC, perceiving parents as being extremely critical), parental expectations (PE, perceiving that one's parents set high goals and expectations on one's performance), personal standards (PS, striving for high standards and goals which important for self-evaluation) and organization (O, importance of order and neatness) (21). These six subscales demonstrate positive and negative dimensions of this construct. Hewitt and Flett (22) subsequently created the Hewitt Multidimensional Perfectionism Scale (MPS) (22). It consists of three subscales: self-oriented perfectionism (SOP, unrealistic high expectations for oneself), other-oriented perfectionism (OOP, unrealistic high expectations for others) and socially-prescribed perfectionism (SPP, the belief that others have high expectations related to oneself to be perfect). Factor analysis was used to analyse these two perfectionism scales, and consistently two main factors of perfectionism were differentiated by researchers: maladaptive evaluation concerns (EC, negative aspects of perfectionism, concerning on failure and mistakes and other's evaluation) and positive achievement striving (PS, adaptive aspects of perfectionism, high expectations, experience of successful performance) (23) (Table 1). Others mentioned the same two main dimensions as personal standards perfectionism (PSP, similar to PS it means the setting of high standards, expectation for oneself) and evaluative concerns perfectionism (ECP, similar to EC it means extremely high critical evaluation for oneself and concerns related to others criticism) (25), or simply adaptive (positive striving) and maladaptive (evaluative concerns) perfectionism (24).

**Table 1:** Two main factors of perfectionism (23, 24).

**Measures of Perfectionism**	**Maladaptive Evaluation Concerns (Maladaptive Perfectionism)**	**Positive Achievement Striving (Adaptive Perfectionism)**
Frost Multidimensional Perfectionism Scale (FMPS)	Concern over mistakes (CM)	Personal standards (PS)
Frost Multidimensional Perfectionism Scale (FMPS)	Parental criticism (PC)	Organization (O)
Frost Multidimensional Perfectionism Scale (FMPS)	Parental expectations (PE)	
Frost Multidimensional Perfectionism Scale (FMPS)	Doubts about actions (DA)	
Hewitt Multidimensional Perfectionism Scale (MPS)	Socially-prescribed perfectionism (SPP)	Self-oriented perfectionism (SOP)
Hewitt Multidimensional Perfectionism Scale (MPS)		Other-oriented perfectionism (OOP)

Perfectionistic concern is associated more strongly with negative outcomes, and perfectionistic striving is characterized by more positive affect, conscientiousness, life satisfaction and achievement (23, 26). However, according to meta-analyses both positive and negative perfectionism can result in psychological distress (27) and psychopathology (28). The effect of positive perfectionism is not perfectly clear, because perfectionistic strivings can have both adaptive and maladaptive consequences (27). Moreover, positive and negative perfectionism do not occur separately; there is an interaction between them, and they can reinforce one another (28–30). Different combinations between the interaction of positive and negative perfectionism have a variety of influences on psychopathology (31, 32).

Problematic perfectionism is highly prevalent among children and young people (23–41%) (33–36). As perfectionistic people want to seem perfect, they tend not to seek help when it is required and they hide their intrapersonal sensitivity, vulnerability and their true pain (37).

Previous literatures emphasizes the importance of childhood and adolescence years in the development of perfectionism (38–43) and NSSI (3, 44–47). Both NSSI and perfectionism are associated with cognitive-affective deficits (1, 27, 48, 49). A large body of evidence suggests that several internalizing and externalizing mental disorders are associated both with NSSI (6, 50–53) and perfectionism (24, 28, 31, 54–63). Furthermore, NSSI is a huge risk factor for suicidal ideation and behavior (28, 51, 64–68), and perfectionism has also a relationship with suicide (28, 67, 68). Both phenomena mean huge concerns related to mental health and psychopathology (1, 28).

Perfectionistic people have a tendency of overconcern for mistakes, doubting related to the ability of appropriate performance and tend to react with strong negative emotions (e.g., shame, guilt) in case of failure in their results (21, 69). Perceived failure can maintain their negative self-evaluation (21), and self-destructive feelings can increase the likelihood of NSSI (4). Self-criticism has a strong relationship with NSSI (70), and emotion regulating function and self-punishment are the most common functions of NSSI behavior (18). Previous studies emphasize that shame may have a key role in NSSI engagement (71, 72), and shame regulation has strong relationship with affect regulation and self-punishment function of NSSI (71, 72). Claes et al. (73) also found that maladaptive perfectionism (evaluative concerns perfectionism) associated with self-punishment, self-torture, and cry-for-help functions of NSSI.

Separately related to NSSI and perfectionism there are available many previous research studies, but only a few investigated the relationship between the two phenomena. More studies suggest that there is an association between NSSI and perfectionism (73–75), but some contain inconsistent findings related to this relationship (73, 76). In recent years the number of research works that have reported results related to the association of perfectionism and NSSI has grown, but till now to the best of our knowledge, no systematic review has been done on this topic. Therefore, we performed a systematic literature review, and the main aim of the study was to include every published study that explored the association between NSSI and perfectionism with both cross-sectional and longitudinal design, without any restrictions on participants age, gender, race or ethnicity in order to explore relationship between two phenomena and clarify the nature of this relationship to improve effective prevention initiatives and treatment strategies. To best of our knowledge, this is the first systematic review to explore relationship between two phenomena. Because of the great conceptual heterogeneity of self-injury definitions (20), not only “NSSI” as a search word was used in our study, but also other search words for self-injurious behavior. We primarily focused on direct, deliberate self-harm without suicidal intent; therefore, we included only those studies in which the meaning of self-injurious behavior definition was similar to the NSSI definition.

The aim of this study is to identify: (a) terminology and definitions of self-injurious behavior, (b) methods for measuring NSSI and perfectionism and (c) the association between NSSI and perfectionism.

## Methods

**Figure 1:**
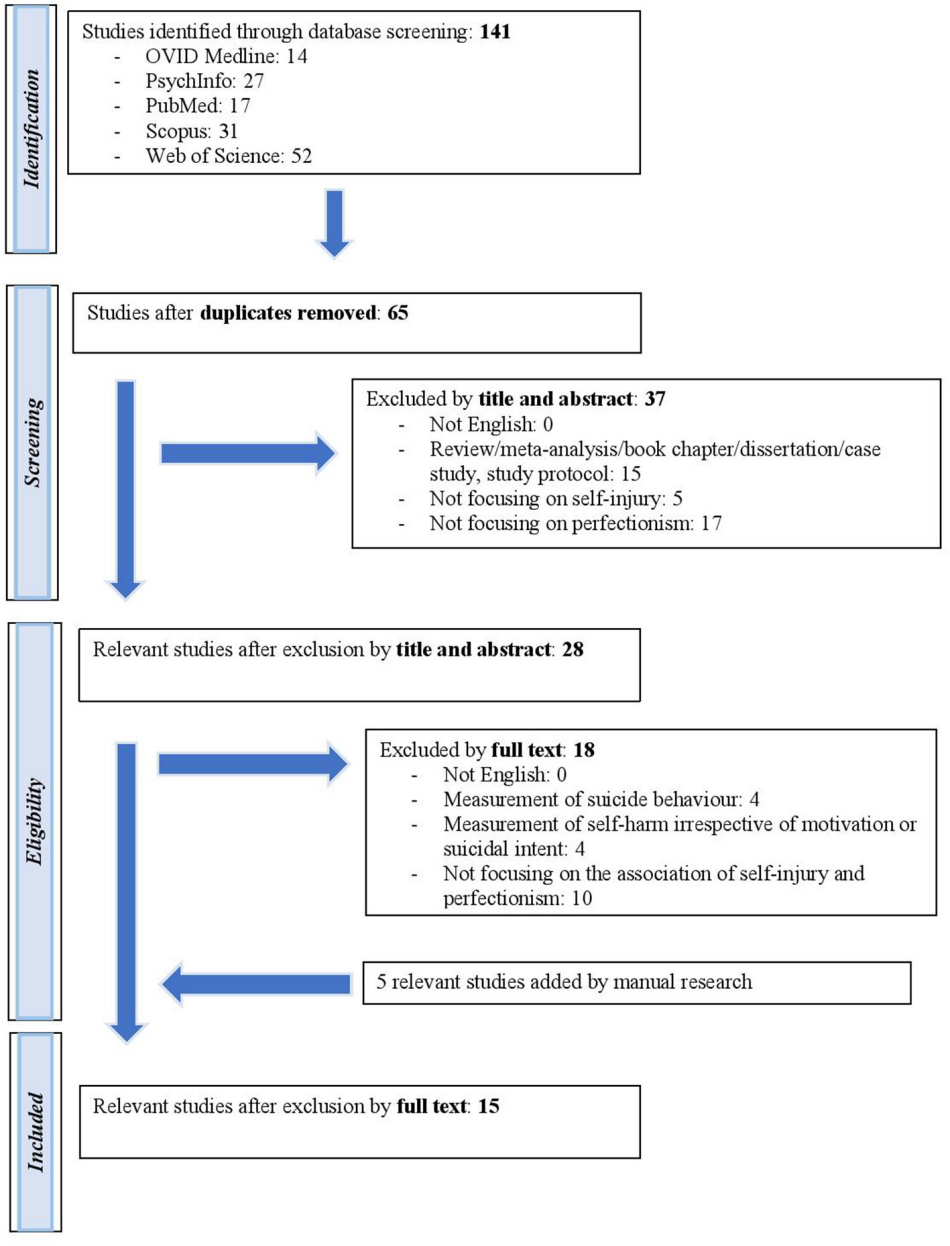
PRISMA flowchart of inclusion-exclusion process.

The methodology of this systematic descriptive review follows the PRISMA guidelines (77). We performed a systematic review of the literature on 31 January 2021 using the following scientific electronic databases: OVID Medline, OVID PsychInfo, PubMed, Scopus, and Web of Science. As search terms we used: (“nonsuicidal self-injury” OR “nonsuicidal self-injury” OR NSSI OR “self-injury” OR “self-injurious behavior” OR SIB OR “self-harm” OR “deliberate self-harm” OR DSH) AND (perfectionism). The full electronic search strategy and search terms were discussed by both authors, and an electronic search was conducted by the first author. The EndNote 20 software program was used to manage the systematization of the papers. For inclusion, studies had to: (a) be written in English, (b) reported empirical data, (c) used validated self-report measures to assess self-injurious behavior and perfectionism and (d) investigate the association of nonsuicidal self-injury and perfectionism. There were no restrictions on participants regarding age, gender, race or ethnicity. Exclusion criteria were studies that (a) that were not written in English, (b) were reviews or meta-analyses, (c) measured suicidal behavior, (d) measured self-injury irrespective of motivation or suicidal intent and (e) were not about the association between nonsuicidal self-injury and perfectionism. Duplicates were searched both automatically by EndNote software and also manually by reading authors and titles again. Articles were first screened by title and abstract by the first author. Of the remaining studies, the full text of articles was read and inspected by the first author. The second author reviewed and supervised the whole screening process. The final selection of relevant studies was verified by both authors according to inclusion and exclusion criteria. Disagreements were resolved by discussion between the authors, and the inclusion or exclusion of the paper was decided by consensus. The final decision related to relevant studies was made by the two authors. A PRISMA flowchart of the selection process is summarized in Figure 1. Two authors assessed independently the methodological quality assessments of included studies (**Table 3**) according to Newcastle-Ottawa Scale (78). Disagreements between the authors were resolved by discussion.

## Results

We found 14 papers in OVID Medline, 27 papers in OVID PsychInfo, 17 papers in PubMed, 31 papers in Scopus and 52 papers in Web of Science, making a total of 141 studies, including duplicates. Furthermore, five papers were added by manual search. After excluding duplicate articles, and after the remaining articles has been checked by inclusion and exclusion criteria, a total of 15 papers were included in our systematic descriptive review (Figure 1, Table 2). Table 3 contains the quality assessment of the included papers.

**Table 2:** Included relevant articles (*N* = 15) examining association between nonsuicidal self-injury and perfectionism.

**References**	**Country**	**Design**	**Age Group**	**No. of Participants**	**Terminology for Self-Injurious Behavior**	**Measurement of Self-Injurious Behavior**	**Measurement of Perfectionism**	**Main Results**
Nock and Prinstein (79)	USA	Cross-sectional, clinical sample, psychiatric inpatient	Age range: 12–17 years, *M* = 14.7 (*SD* = 1.4)	*N* = 89 (23 boys, 66 girls 74.15%) psychiatric inpatients, adolescent	Self-mutilative behavior (SMB)	Functional Assessment of Self-Mutilation (FASM)	Child and Adolescent Perfectionism Scale (CAPS)	Socially prescribed perfectionism was related to the social negative and social positive reinforcement functions of SMB. Self-oriented perfectionism had no relationships with SMB functions.
Yates et al. (47)	USA	Cross-sectional and longitudinal, community sample	n.a.	Cross-sectional sample of 9–12th graders (*N* = 1, 036, 51.9% girls, 538 girls, 498 boys), longitudinal sample from the 6th through 12th graders: (*N* = 245, 53.1% girls, 130 girls, 115 boys).	NSSI	Functional Assessment of Self-Mutilation (FASM)	Parental criticism scale from the Frost Multidimensional Perfectionism Scale (FMPS)	Parental criticism predicts NSSI in both cross-sectional and the longitudinal samples. Youth alienation toward parents emerged as a relevant process underlying this pathway.
Hoff and Muehlenkamp (80)	USA	Cross-sectional, community sample	*M* = 19.82 years (*SD* = 2.86)	*N* = 165 undergraduate students (24.7% male, 75.3% female) (56 with a history of NSSI and *N* = 109 control subjects with no history of NSSI)	NSSI	Deliberate Self-Harm Inventory (DSHI)	Frost Multidimensional Perfectionism Scale (FMPS)	Participants with NSSI significantly differ from no-NSSI participants on three perfectionism subscales. Individuals with NSSI reported significantly higher score on concern over mistakes and parental criticism and significantly lower on organization subscales of FMPS.
Claes et al. (73)	Belgium	Cross-sectional, clinical sample, ED patient	Age range: 14–42 years, *M* = 21.5 years (*SD* = 6.23)	*N* = 95 ED patients (women)	NSSI	Self-Injury Questionnaire (SIQ)	Frost Multidimensional Perfectionism Scale (FMPS)	ED patients with NSSI reported significantly higher levels of parental criticism and evaluative concerns perfectionism (ECP) compared with ED patients without NSSI. ECP was positively related to the self-punishment, self-torturing and cry-for-help functions of NSSI. PC was negatively related to cry-for-help function of NSSI. ECP was found to mediate the association between parental criticism and NSSI symptoms. There was no relationship between PSP and NSSI.
Fujimori et al. (74)	Japan	Cross-sectional, both clinical and community sample	ED + SIB sample: *M* = 24.3 years (*SD* = 5.6), ED + no SIB sample: *M* = 26.9 years (*SD* = 7.9), healthy control group: *M* = 19.5 years (*SD* = 1.2)	Clinical sample: *N* = 80 female ED patients (ED + SIB: *n* = 25, ED+ no SIB: *n* = 55), healthy control sample: *N* = 120 female university students	Self-injurious behavior (SIB)	SIB were established through own developed questions.	Perfectionism subscale from the Eating Disorder Inventory	Perfectionism score was significantly higher for the ED + SIB group compared to the ED/no SIB and control groups.
Flett et al. (81)	Canada	Cross-sectional, community sample	*M* = 18.89 years (*SD* = 2.30)	*N* = 319 university students (112 men, 207 women 64.9%)	Deliberate self-harm (DSH)	Deliberate Self-Harm Inventory (DSHI), Self-Harm Inventory (SHI)	Multidimensional Perfectionism Scale (MPS), The Frost Multidimensional Perfectionism Scale (FMPS)	Increased self-harm in men had negative relationship with other-oriented perfectionism. Increased self-harm in women was related to increased parental criticism and SPP.
Miskey et al. (82)	USA	Cross-sectional, community sample	*M* = 19.10 years (*SD* = 2.05)	*N* = 292 undergraduate students (62.3% women)	NSSI cutting	Deliberate Self-Harm Inventory (DSHI)	Perfectionism Inventory (PI)	NSSI cutting duration was associated positively with perfectionistic rumination. NSSI cutting onset age was positively correlated with concern over mistakes, and need for approval scales of PI. Frequency of NSSI cutting was predicted by perfectionistic rumination, organization, and low concern over mistakes (accounting for 31% of the variance).
Luyckx et al. (75)	Belgium	Cross-sectional, both clinical and community sample	Community sample: *M* = 15.95 years (*SD* = 1.30), psychiatric sample: *M* = 28.09 years (*SD* = 9.84)	Community sample: *N* = 348 female high school students, psychiatric sample: *N* = 131 female psychiatric patients (80 ED, 51 BPD)	NSSI	Self-Injurious Questionnaire-Treatment Related (SIQ-TR)	Perfectionism subscale from the Eating Disorder Inventory-2	According to hierarchical logistic regression analysis, perfectionism was associated with a significantly greater likelihood of engaging in NSSI in female adolescent sample.
Eichen et. al. (83)	USA	Cross-sectional, community sample	Age range 18–25 years, *M* = 20.61 years (*SD* = 1.97)	Community sample, women (*N* = 508)	NSSI	Functional Assessment of Self-Mutilation (FASM)	Perfectionism subscale from the Eating Disorder Inventory-2	There was no significant difference between the score of perfectionism across four groups: no NSSI/Suicidal Ideation, NSSI-only, Suicidal Ideation-only, and NSSI/Suicidal Ideation.
Varela-Besteiro et al. (84)	Spain	Cross-sectional, clinical sample	Age range 12–17 years, *M* = 14.74 years (*SD* = 1.53)	Adolescents with ED, *N* = 109; 87.2% female (*n* = 95), 12.8% (*n* = 14) male.	NSSI	Based on clinical interview	Child and Adolescent Perfectionism Scale (CAPS)	NSSI group of ED patients had significantly higher scores as compared to the non-NSSI ED group (without self-injurious behavior) on all EDI-2 perfectionism scales and on the CAPS total score.
Kaur and Martin (85)	Australia	Cross-sectional, community sample	Age range: 19–36 years, *M* = 23.1 years (*SD* = n.a)	Postgraduate medical students, *N* = 260 (139 males and 121 females, 46.5%)	NSSI	Deliberate Self-Injury Questionnaire	Frost Multidimensional Perfectionism Scale (FMPS)	Participants with NSSI compared to those without NSSI reported higher scores on perfectionism total score, on parental expectations, on concern over mistakes, on doubts about action. The largest effect has concern over mistakes. Maladaptive perfectionism was also significantly higher in NSSI group.
Vieira et al. (76)	Portugal	Cross-sectional, clinical sample	Age range: 14–49 years, *M* = 22.12 years (*SD* = 6.31)	ED female outpatients, *N* = 245	NSSI	Oxford Risk Factor Interview for Eating Disorder	Oxford Risk Factor Interview for Eating Disorder: subject's mental health domain—perfectionism subdomain.	There is no relationship between perfectionism and NSSI among ED patients.
Lucas et al. (86)	USA	Cross-sectional, community sample	Age range: 18-46 years, *M* = 19.6 years (*SD* = 3.12)	*N* = 386 college students (267 females 69, 2%, 116 males 30, 8%)	NSSI	The Self-Harm Inventory (SHI)	Frost Multidimensional Perfectionism Scale (FMPS)	FMPS dimensions (concern over mistakes, parental expectation, parental criticism, doubts about action) were positively associated with NSSI. According to results of hierarchical regression analysis, perfectionism (concern over mistakes dimension) is a significant predictor of NSSI.
Chang et al. (87)	USA	Cross-sectional, community sample	Age range: 18–25 years, *M* = 20.2 years (*SD* = 1.61)	*N* = 287 women college students	NSSI	The Self-Harm Inventory (SHI)	Frost Multidimensional Perfectionism Scale (FMPS)	Perfectionism was found to predict additional unique variance in NSSI, even after accounting for sexual assault history. Evaluative concerns dimension is the most consistent unique predictor of NSSI.
Newman et al. (88)	USA	Cross-sectional, community sample	Age range: 18–27 years, *M* = 18.9 (*SD* = 1.19)	*N* = 410 undergraduate psychology students (77.1% female)	Self-harm	One item from the Nonsuicidal Self-Injury Assessment Tool (NSSI-AT)	Perfectionism Inventory (PI), Perfectionistic Cognitions Inventory (PCI)	With principal components analysis four different profiles of perfectionism were defined: obsessive, constructive, non-perfectionist, motivated. Profiles of perfectionism were significantly associated with differences in self-harm.

**Table 3:** Quality assessment of the included studies.

**References**	**Selection**	**Comparability**	**Exposure**
Nock and Prinstein (79)	**		*
Yates et al. (47)	**		*
Hoff and Muehlenkamp (80)	****	**	**
Claes et al. (73)	***	**	*
Fujimori et al. (74)	****	**	**
Flett et al. (81)	**		*
Miskey et al. (82)	**		*
Luyckx et al. (75)	****	**	**
Eichen et al. (83)	****	**	**
Varela-Besteiro et al. (84)	***	**	**
Kaur and Martin (85)	****	**	**
Vieira et al. (76)	***	**	**
Lucas et al. (86)	**		*
Chang et al. (87)	**		*
Newman et al. (88)	**		*

### Description of the Studies Selected

Table 2 provides a summary of the data obtained from each study. All 15 studies were published in the last 16 years-−12 of them were brought out in the last 10 years, and 7 of the 15 studies were written in the last 5 years (76, 83–88).

Regarding to geographical distribution, eight of the 15 studies (53.3%) reported data from the United States (47, 79, 80, 82, 83, 86–88), while one was Canadian (84) and four were (26.7%) European, containing data form Belgium (two papers) (73, 75), Spain (one paper) (83) and Portugal (one paper) (76). There was also one study form Japan (74), and one form Australia (85) were involved in our systematic review.

Altogether, nine of the 15 studies (60%) recruited participants from community samples (47, 80–83, 85–88), four (26.7%) enrolled clinical populations (73, 76, 79, 84), and two studies (13.3%) represent data from both community and clinical participants (74, 75). Yates et al. (47) investigated two different community samples. One was recruited from the local community and schools for a cross-sectional investigation; the other sample was a New England Study of Suburban Youth (NESSY) cohort for a longitudinal investigation.

Six studies (40%) enrolled college, undergraduate students, whose mean ages were between 18.89 and 20.61 years (74, 80–83, 87). Four (26.7%) studies recruited adolescents with a mean age range of 14.7–15.95 years (47, 75, 79, 80). A further six studies recruited a wider age range, form 14-49 years, including adolescent and adult participants (73, 75, 76, 85, 86, 88). Yates et al. (47) enrolled adolescents from 9 to12^th^ graders for their cross-sectional studies, and for a longitudinal sample children were recruited from the 6th grade and were followed annually through the 12th grade. Other than school grade information, the mean ages of included children and adolescents are not reported in the paper.

Regarding gender distribution, six (40%) papers included only female participants (73–76, 83, 87), and the other nine involved studies (60%) investigated both female and male populations (47, 79–82, 84–86, 88). Of these nine studies, seven investigated mainly female participants (62.3–87.2% in the examined population), and only two studies contained an approximately balanced male-female ratio population (47, 85).

Considering the study design, 14 (93.3%) were cross-sectional studies, while one consisted of a cross-sectional and longitudinal investigation (47).

### Terminology and Definitions of Self-Injurious Behavior Related to Relevant Included Studies

Because of the great conceptual heterogeneity of self-injury definitions, we have summarized how the definition of self-injurious behaviors was used in the included studies.

Altogether, five different terms of self-injurious behavior were mentioned in the 15 investigated papers. All 15 defined and measured self-injurious behavior as a nonsuicidal act (47, 73–76, 79–88).

Of the 15 studies selected, 10 (66.7%) represented studies on NSSI (47, 73, 75, 76, 80, 82, 83, 85–87). The term “NSSI” was the one most commonly used, and this term was used similarly to the official definition (5). NSSI is a physical self-injurious behavior, although Varela-Besteiro et al. (84) used the term of self-inflicted physical harm, including also a drug overdose without suicidal intent. According to this term, they use NSSI as an expression related to self-injurious behavior.

Newman et al. (88) use “self-harm,” but they did not give a definition. However, in the chapter on measurement can be found the question “Have you ever hurt your body on purpose but without wanting to end your life?,” which shows how they explored self-injurious behavior.

Flett et al. (81) define “deliberate self-harm” (DSH) as intentional self-injury without suicidal intent. Beyond direct physical self-harm, they consider self-harm behavior to include substance abuse or putting oneself in a dangerous situation.

Fujimori et al. (74) use the term of “self-injurious behavior” (SIB) in a nonsuicidal meaning, similar to the official NSSI definition, as direct and deliberate physical damage of one's body surface.

Nock and Prinstein (79) use “self-mutilative behavior” (SMB). It is also used in a nonsuicidal meaning as direct physical damage of one's body.

All the included papers in this study apply the term of self-injurious behavior with nonsuicidal meaning of these acts.

### Measurement of Self-Injurious Behaviors in the Included Studies

Among the 15 papers involved, both diagnostic interviews and self-reported questionnaires were used to measure self-injurious behaviors. Altogether, eight different instruments were used.

Self-report questionnaires were applied for measuring self-injurious behaviors in 12 studies (47, 73, 75, 79–83, 85–88). The Functional Assessment of Self-Mutilation (FASM) (89) was used in three studies (47, 79, 83). Over the previous 12 months it enables us to assess the frequency of different methods of SMB, the degree of physical pain, the use of alcohol or drugs during SMB, the amount of time about the incident before engaging and the awareness of this behavior by friends. The Deliberate Self-Harm Inventory (DSHI) (17) appears in three studies (80–82). It assesses with 17-items the different types of DSH. This instrument enables us to evaluate also the frequency, severity and duration of self-harm acts. With it, Miskey et al. (82) focused only on NSSI cutting. The Self-Injury Questionnaire (90) was applied in the study of Claes et al. (73) to measure self-injury in the previous 12 months by means of hair pulling, scratching, bruising, cutting and burning. It also enables us to assess the age of onset, the frequency, the function of self-injurious behavior, pain, emotional experiences during engagement and the injured body part. Luyckx et al. (75) used the Self-Injurious Questionnaire-Treatment Related (SIQ- TR) (91). It measures the type, frequency, duration, age of onset, emotional experiences before and after of engagement, injured body part and functions of self-injury. The Self-Harm Inventory (SHI) (92) was mentioned in three studies (81, 86, 87). It was used to assess with 22 items the history of self-harm, ranging from eating disorder-specific actions (exercised an injury on purpose) to those related to medical concerns (e.g., not allowing a wound to heal). Flett et al. (81) measured self-harm with 22 items, using questions drawn from the DSHI (17) and SHI (92). Newman et al. (88) used one item (“Have you ever hurt your body on purpose but without wanting to end your life?”) from the Nonsuicidal Self-Injury Assessment Tool (NSSI-AT) (93). The Deliberate Self-Injury Questionnaire (94) was mentioned in the study of Kaur and Martin (85). They measured the frequency, purpose, types and cessation of self-injurious behavior.

Vieira et al. (76) applied the Oxford Risk Factor Interview (RFI) (95). This semi-structured interview assesses the putative risk factors of eating disorders patient and focuses on the period before the onset of eating pathology. They used three questions in connection with occurrence, type and frequency.

Varela-Besteiro et al. (84) measured self-injurious behavior during clinical interviews.

Fujimori et al. (74) used their own developed questions related to occurrence, injured body-part and degree of felt pain during acts.

### Measurement of Perfectionism in the Included Studies

The majority of papers (14 studies from the 15 ones) used self-reported questionnaires in order to assess perfectionism, while one study used interviews. Six different instruments were used in the included studies.

The most frequently used questionnaire was the FMPS (21). It was mentioned in seven studies (47, 73, 80, 81, 85–87). It measures with 35 items the adaptive and maladaptive dimensions of perfectionism with six different subscales. Hoff and Muehlenkamp (80) and Lucas et al. (86) measured multiple aspects of perfectionism with all 35 items of the FMPS (six subscales). Yates et al. (47) used only the PC subscale form the FMPS with four items. Claes et al. (73) and Chang et al. (87) measured two factors: maladaptive perfectionism or ECP (containing the CM, DA subscales) and adaptive perfectionism or PSP (containing the PS subscale) (25). In addition, Claes et al. (73) also used the PC scale. After conducting factor analyses, Kaur and Martin (85) used the FMPS-29 (96) with five subscales (CM, DA, PE/PC, O, PS).

Flett et al. (81) used those versions of the FMPS that did not include the O factor (97). In addition, they also measured the level of SOP, OOP and SPP with MPS (22).

The Child and Adolescent Perfectionism Scale (CAPS) (98) was applied in two studies (79, 84). It measures multiple dimensions of perfectionism with two subscales (self-oriented, socially prescribed perfectionism) with 22-items.

In three studies (74, 75, 83), perfectionism was assessed with the perfectionism subscale of the Eating Disorders Inventory (EDI) (99). It measures perfectionism only unidimensionally without differentiation of negative and positive aspects.

The Perfectionism Inventory (PI) (100) was applied in two studies (82, 88). It enables us to measure perfectionism with eight scales: organization, striving for excellence, planfulness, high standards for others, concern over mistakes, need for approval, rumination, and perceived parental pressure.

Newman et al. (88) used the Perfectionistic Cognitions Inventory (PCI) (101), which assesses with 25 items the automatic thoughts related to the need to be perfect.

Vieira et al. (76) used the RFI (95) to measure self-injury as well as perfectionism. To assess perfectionism, participants were asked, “If you go back to your adolescence and childhood, did you have very high goals and demands at work/school and in other areas, more than other people your age? Would you be angry if you did not meet these goals and demands?”

### Association Between NSSI and Perfectionism

In this section we summarize results related to the association between NSSI and perfectionism separately in clinical and community samples. We also summarize the comparison between clinical and control groups.

### Association in Clinical Samples

Nock and Prinstein (79) measured the features and functions of SMB among 89 adolescent psychiatric inpatients (74.1% girls, 12–17 years, *M* = 14.7, *SD* = 1.4). Four SMB functions were measured: automatic negative reinforcement, automatic positive reinforcement, social positive reinforcement and social negative reinforcement in connection with socially prescribed and self-oriented perfectionism. According to their results, socially prescribed perfectionism was associated with SMB social negative reinforcement functions (β = 0.23, *p* < 0.001) and with SMB social positive reinforcement functions (β = 0.30, *p* < 0.01). Self-oriented perfectionism had no relationships with the measured SMB functions.

Claes et al. (73) examined the difference between female ED patients (*N* = 95, age range: 14–42 years, *M* = 21.5 years, *SD* = 6.23, 38.9% with NSSI) with and without NSSI related to PSP and ECP. ED patients with NSSI have significantly higher scores on ECP [*F*_(3, 83)_ = 5.58, *p* < 0.05] and PC [*F*_(3, 83)_ = 5.62, *p* < 0.05] compared to ED patients without NSSI. Linear regression analyses showed a significant positive association between the self-punishment (β = 0.30, *p* < 0.05) and self-torturing (β = 0.30, *p* < 0.05) and cry-for-help (β = 0.36, *p* < 0.01) functions of NSSI and ECP. In addition, the cry-for-help function of NSSI has a negative relation with PC (β = −0.29, *p* < 0.01). According to regression analysis, the effect of PC on NSSI was significant (β = 0.28, *p* < 0.01). However, this initial association between PC and NSSI turned to non-significant after taking into account ECP, and the relationship between PC and NSSI was only indirect through a mediation effect of ECP (PC-ECP association: β = 0.34, *p* < 0.001; ECP-NSSI association: β = 0.22, *p* < 0.05). According to results, there was no relationship between PSP and NSSI.

Varela-Besteiro et al. (84) explored in adolescents ED patients (*N* = 109, 87.2% female, 12.8% male, *M* = 14.74 years, *SD* = 1.53) the association between self-injurious behavior and suicidal thoughts, and symptoms of depression, anxiety, motivation for change and perfectionism. According to the presence of self-injurious behavior, they defined two groups: an NSSI group (*n* = 34), and a non-NSSI group (*n* = 75). The NSSI group of ED patients had significantly higher scores compared to the non-NSSI ED group on EDI-2 perfectionism scales (NSSI group: *M* = 6.91, *SD* = 3.69; non-NSSI group: *M* = 4.51, *SD* = 4.24; Mann-Whitney U-test = 779.50, *p* = 0.001), and on the CAPS total score (NSSI group: *M* = 70.29, *SD* = 11.89; non-NSSI group: *M* = 63.55, *SD* = 15.86; Mann-Whitney U-test = 945.50, *p* < 0.05).

Vieira et al. (76) explored the potential risk factors for NSSI among female ED patients (*N* = 245, *M* = 22.12, *SD* = 6.31). There was no significant difference between the non-NSSI group (*n* = 156, 67%) and the NSSI group (*n* = 77, 33%) related to perfectionism. Because there was not an initial significant association between perfectionism and the non-NSSI or NSSI group, the perfectionism variable was not involved in further regression analysis.

### Association in Community Samples

Yates et al. (47) explored the pathways between the perceived PC perfectionism dimension and NSSI among 9–12th graders in a cross-sectional sample (*N* = 1,036, 51.9% girls, 538 girls, 498 boys) and in a longitudinal sample (*N* = 245, 53.1% girls, 130 girls, 115 boys) followed from the 6th through 12th grades. According to mediation analysis in the cross-sectional sample among girls, perceived PC had a direct relationship with an increased probability of engaging in NSSI (*B* = 0.11, SEB = 0.02, *p* < 0.05, 95% CI = 0.07, 0.16) but had no association with the frequency of NSSI (*B* = 0.02, SEB = 0.01, *p* > 0.05). When parental alienation was added to the mediation analysis, the direct association between PC and NSSI was no longer significant (*B* = 0.02, SEB = 0.03, *p* > 0.05, 95% CI = −0.03, 0.07), and only an indirect path through parental alienation was significant (PC-parental alienation: *B* = 0.69, SEB = 0.04, *p* < 0.001; parental alienation-probability of NSSI: *B* = 0.15, SEB = 0.02, *p* < 0.001). Among boys in cross-sectional samples, perceived PC had a direct relationship with both probability of NSSI (*B* = 0.08, SEB = 0.03, *p* < 0.05 95% CI = 0.02, 0.13) and with the frequency of NSSI (*B* = 0.07, SEB = 0.02, *p* < 0.01 95% CI = 0.04, 0.11). Similarly, to girls, when parental alienation was involved in the mediation analysis there was only an indirect relationship between PC and the probability of NSSI (PC-parental alienation: *B* = 0.61, SEB = 0.05, *p* < 0.001; parental alienation—probability of NSSI: *B* = 0.12, SEB = 0.03, *p* < 0.001) and between PC and the frequency of NSSI (*B* = 0.61, SEB = 0.05, *p* < 0.001; *B* = 0.07, SEB = 0.03, *p* < 0.05) through parental alienation. Similar to the results of the cross-sectional sample, in the case of the longitudinal sample among girls, perceived PC in grades 6 to 8 has a direct significant relationship with the probability of NSSI in grade 12 (*B* = 0.13, SEB = 0.07, *p* < 0.05 95% CI = 0.01, 0.26). When parental alienation was added to mediation analysis, this direct pathway turned to non-significant (*B* = 0.08, SEB = 0.08, *p* > 0.05 95% CI = −0.08, 0.25). In addition, there was also no indirect relationship between PC and the probability of NSSI through parental alienation (PC-parental alienation: *B* = 0.49, SEB = 0.09, *p* < 0.001; parental alienation—probability of NSSI: *B* = 0.10, SEB = 0.08, *p* > 0.05). In the case of boys, the initial direct effect between PC and the probability of NSSI was not significant (*B* = 0.14, SEB = 0.08, *p* < 0.10), so a mediated model was not examined. In the longitudinal sample the relationship between PC and the frequency of NSSI was not significant in both genders.

Hoff and Muehlenkamp (80) examined the association between NSSI and perfectionism, cognitive rumination, depression and anxiety in 165 college students (56 with NSSI, 109 control, *M* = 19.82 years, *SD* = 2.86). The NSSI group (individuals with a history of NSSI) compared to the controls (individuals without NSSI) reported a significantly higher score on two subscales of the FMPS: CM *F*_(1, 153)_ = 9.58, *p* < 0.01, and PC, *F*_(1, 153)_ = 8.94, *p* < 0.01, and a significantly lower score on the O subscale: F *F*_(1, 153)_ = 18.34, *p* < 0.01. According to the results of a binary logistic regression analysis, the O subscale of the FMPS was negatively significant with NSSI (*B* = −1.94, *SE* = 0.049, Wald Statistic = 15.68, *p* < 0.01).

Flett et al. (81) measured the self-punitiveness model (including perfectionism, overgeneralisation, self-criticism, and shame) as it related to DSH among 319 university students (64.9% female, *M* = 18.89 years *SD* = 2.30). According to the results, in men increased self-harm had a negative relationship with OOP (*r* = −0.40 *p* < 0.01). Increased self-harm in women was associated with increased PC (*r* = 0.20 *p* < 0.01) and with increased SPP (*r* = 0.16 *p* < 0.05).

Miskey et al. (82) explored the role of the Big Five personality dimension and perfectionism in predicting nonsuicidal cutting among 292 undergraduate students (62.3% women, *M* = 19.10, *SD* = 2.05). NSSI cutting duration was associated positively with the rumination scale of PI (*r* = 0.29 *p* < 0.05). The onset age of NSSI cutting was positively correlated with the PI scales of concern over mistakes (*r* = 0.25 *p* < 0.05) and need for approval (*r* = 0.24 *p* < 0.05). According to multiple regression analyses, perfectionism variables rumination (β = 0.809, *p* < 0.05), concern over mistakes [negatively weighted, β = −0.784, *p* < 0.01) and organization (β = 0.452, *p* < 0.05)] predict statistically significant the NSSI cutting frequency (*F* = 2.324, *p* = 0.037), and accounting for 31% of the variance.

Eichen et al. (83) examined the association between depression, anxiety, and stress and eating disorder-specific psychopathology among college-aged women (*N* = 508, *M* = 20.61 years, *SD* = 1.97) with and without NSSI and with or without suicidal ideation. They divided all participants into four groups: no NSSI/suicidal ideation (*n* = 400, 78.7%), NSSI-only (*n* = 70, 13.8%) suicidal ideation-only (*n* = 25, 4.9%) and NSSI + suicidal ideation (*n* = 13, 2.6%). The perfectionism score did not differ significantly across groups: no NSSI/suicidal ideation (*M* = 4.09, *SD* = 1.15), NSSI-only (*M* = 4.24, *SD* = 1.10), suicidal ideation-only (*M* = 4.21, *SD* = 1.19) and NSSI + suicidal ideation (*M* = 4.22, *SD* = 1.23); *F*_(3, 503)_ = 0.43; *p* = 0.74, partial η^2^ = 0.003.

Kaur and Martin (85) examined nonsuicidal self-injury among medical students (*N* = 260, 139 males (53.4%), 121 females, *M* = 23.1 years, *SD* = n.a.) and its relationship with level of perfectionism. Participants with NSSI compared to those without NSSI reported higher scores on the perfectionism total score: *F*_(1, 258)_ = 9.21, *p* < 0.01; on PE: *F*_(1, 258)_ = 3.92, *p* < 0.01; on CM: *F*_(1, 258)_ = 9.16, *p* < 0.01; on DA: *F*_(1, 258)_ = 4.96, *p* < 0.05. The largest effect was concern over mistakes. Maladaptive perfectionism was also significantly higher in the NSSI group: *F*_(1, 258)_ = 12.85, *p* < 0.001.

Lucas et al. (86) examined the association between perfectionism, social problem solving, and NSSI among college students (*N* = 386, 69.2% female, *M* = 19.6 years, *SD* = 3.12). Four FMPS dimensions were associated positively with NSSI: CM (*r* = 0.24 *p* < 0.001), PE (*r* = 0.12 *p* < 0.05), PC (*r* = 0.18 *p* < 0.001) and DA (*r* = 0.18 *p* < 0.001). According to results of a hierarchical regression analysis before controlling for suicide risk, perfectionism accounted for 9% of the variance in NSSI [*F*_(6, 321)_ = 5.56, *p* < 0.001], and after controlling for suicide risk, perfectionism accounted smaller 3% of variance in NSSI [*F*_(6, 320)_ = 2.33, *p* < 0.05]. Within perfectionism, only the CM dimension was a significant predictor for NSSI before (β = 0.31, *p* < 0.001) and also after (β = 0.22, *p* < 0.01) controlling for suicide risk. It should be mentioned that when social problem solving was entered in the model of regression analysis before perfectionism, the effect of perfectionism before [*F*_(6, 316)_ = 1.83, *p* > 0.05] and also after controlling for suicide risk [*F*_(6, 315)_ = 1.05, *p* > 0.05] and CM dimension turned to non-significant (β = 0.14, *p* > 0.05), and it did not predict NSSI significantly.

Chang et al. (87) examined the relationship between sexual assault history and perfectionism (positive strivings, evaluation concerns dimensions) with NSSI and suicidal behaviors in women college students (*N* = 287, ages ranged from 18 to 25 years, *M* = 20.2 years, *SD* = 1.61). According to results, positive strivings have a positive relationship with evaluative concerns (*r* = 0.36, *p* < 0.001). The PS subscale correlated significantly with DA (*r* = 0.19 *p* < 0.001) and CM (*r* = 0.40, *p* < 0.001) subscales. Results of a hierarchical regression analysis show that perfectionism was found to account for 4% of variance in NSSI behavior [*F*_(2, 281)_ = 6.22, *p* < 0.01). Within perfectionism, evaluative concerns predicted NSSI significantly (β = 0.21, *p* < 0.001). The interaction term of personal strivings x evaluative concerns had no significant effect on the variance of NSSI *F*_(1, 280)_ = 0.08, *p* > 0.05). After controlling the overlap between NSSI and suicidal behaviors, perfectionism was found to account for a marginally significant 1% of variance in NSSI behaviors *F*_(2, 280)_ = 2.67, *p* < 0.10). Only the evaluative concerns dimension was a significant predictor for NSSI behavior (β = 0.13, *p* < 0.05). In this case the interaction term of personal strivings x evaluative concerns also had no significant effect on the variance of NSSI *F*_(1, 279)_ = 0.41, *p* > 0.05).

Newman et al. (88) examined emotions, cognitions and behaviors related to healthy and unhealthy expressions of perfectionism among undergraduate students (*N* = 410, *M* = 18.9 years, *SD* = 1.19). Based on six factors of perfectionism which were identified (rumination, planfulness, drive, academic management, compulsion and emotional suppression) by submitting measures of behavioral, emotional, and cognitive correlates with principal components analysis, they set four different profiles of perfectionism: obsessive (*n* = 84), constructive (*n* = 87), non-perfectionist (*n* = 122) and motivated (*n* = 117). Profiles of perfectionism were significantly associated with differences in self-harm [*F*_(3, 402)_ = 2.85, *p* = 0.037]. Mean scores related to self-harm for profiles: obsessive (*M* = 0.33), constructive (*M* = 0.11), non-perfectionist (*M* = 0.28), motivated (*M* = 0.25). Obsessive perfectionists have the worst mental health, with high rumination, planfulness and compulsion tendency.

### Comparison of the Association Between Clinical and Community Samples

Fujimori et al. (74) evaluated the relationship among parental bonding, ED and SIB among 80 female ED patients (*M* = 24.3 years, *SD* = 5.6) and 120 healthy female university control students (*M* = 19.5 years, *SD* = 1.2). According to their results, the ED + SIB group (*n* = 25) reported a significantly higher score on perfectionism (*M* = 9.7, *SD* = 4.1) than ED/no SIB (*n* = 55) (*M* = 7.0, *SD* = 4.6) and control groups (*n* = 120) (*M* = 4.3, *SD* = 4.1) [*F*_(2, 197)_ = 20.6, *p* < 0.001, *post-hoc* test: ED+SIB > ED/no SIB > control].

Luyckx et al. (75) examined whether identity process predicted NSSI above and beyond anxiety, depression, Big Five personality traits, perfectionism and effortful control. They involved 348 female adolescents (*M* = 15.95 years, *SD* = 1.30) and 131 female psychiatric patients (80 ED and 51 BPD) (*M* = 28.09 years, *SD* = 9.84). Spearman Rho's correlation coefficient shows that engaging in NSSI was positively related to perfectionism in both samples (*r* = 0.25, *p* < 0.01). Hierarchical logistic regression analyses demonstrated in the female high school adolescents sample that perfectionism was related to a significantly greater likelihood of engaging in NSSI (*B* = 0.85, SE = 0.21, Wald statistic = 16.47, *p* < 0.001). In psychiatric patients, there was no significant association between NSSI and perfectionism (*B* = 0.19, SE = 0.27, Wald statistic = 0.50, *p* > 0.05).

## Discussion

This paper provides an up-to-date overview of the existing literature related to the association between NSSI and perfectionism. To our knowledge, this is the first systematic review on this topic. To perform this systematic review, we involved 15 studies. Our aim was to summarize and clarify the nature of the relationship between two phenomena. Summarizing the results of the included studies, there is a clear relationship between NSSI and perfectionism.

Regarding descriptive findings, the majority of studies (60%) reported data from North America, while 26.7% were from Europe. More than half (60%) of included studies investigated community samples (47, 80–83, 85–88), while about a fourth (26.7%) were enrolled from clinical populations (73, 76, 79, 84); only two of the 15 studies (13.3%) (74, 75) compared both community and clinical samples in connection with self-injury and perfectionism. Although a large body of previous literature emphasizes that several mental disorders, both internalizing and externalizing disorders, are associated with both NSSI (6, 50–53) and perfectionism (24, 28, 31, 54–63), the majority of the clinical samples investigated ED populations (73–76, 84). Almost all the studies had a cross-sectional design and only one study contained both a cross-sectional and longitudinal investigation (47). This draws the attention of researchers to the need of longitudinal studies on this topic that will be able to examine the possible causal relationship between NSSI and perfectionism.

The first NSSI engagement generally occurs in early adolescent years; the typical age of onset is 12–14 years (6), and the peak of NSSI prevalence is around 15–17 years (6, 7). Furthermore, it was found that in late adolescence, NSSI prevalence decreases with age (1). According to previous evidence, the prevalence of NSSI in community adolescents was found to be 17–46.5% (2, 8–10); in clinical samples of adolescent it was 60–80% (11, 12), and among adult community samples it was found to be around 4–23% (2, 13, 14). The importance of childhood and adolescence years related to the development of perfectionism (38–43) and NSSI (3, 44–47) has also been proved. Despite previous evidence in our systematic review, only four studies, or about one quarter of the studies (26.7%), (47, 75, 79, 84) investigated the association between the two phenomena, especially in the adolescent sample. Three of these four studies measured this relationship in a clinical adolescent sample (75, 79, 84) and two of them in ED population (75, 84). Only Nock and Prinstein (79) enrolled adolescent psychiatric inpatients irrespective of psychiatric diagnosis. Altogether, 40% (six of 15) of the studies explored undergraduate students with a mean age between 18.89 and 20.61 years (74, 80–83, 87); another 40 % (again 6 from 15) of the studies involved a population with a wider age range of 14–49 years (73, 75, 76, 85, 86, 88), so the differentiation of results between adolescent and adult participant is not possible.

NSSI behavior is more common among women than in men (15). In our systematic review, the studies examined mainly female participants. Altogether, 40% (six of 15) of papers involved only women participants (73–76, 83, 87), and although in the other 60% (9 from 15) studies the populations were derived both from female and male participants, in seven of the nine studies the proportion of females was much higher than men, between 62.3 and 87.2% (79–82, 84, 86, 88). In spite of the fact that NSSI is more common among women, further studies should have more focus on NSSI behavior in men as well for understanding the reasons.

Related to the terminology of self-injury, five terms were mentioned in the 15 investigated papers. All the papers apply the term of self-injurious behavior with nonsuicidal meaning. Among these terms, NSSI was the most common—it was used in two-thirds (66.7%) of the studies, according to the definition suggested by DSM-5 (5). Although this definition emphasizes that this is a deliberate, direct, nonsuicidal, self-inflicted destruction of body tissue without social acceptance, and does not contain drug overdose (5), Varela-Besteiro et al. (84) used the expression of NSSI related to self-injurious behavior including also drug overdose. The terms of SIB and SMB were used similarly to the NSSI definition, focusing on nonsuicidal physical damage of body surface (74, 79). Flett et al. (81) applied DSH also in a nonsuicidal meaning, but this term contains as well substance abuse and putting self to dangerous situation. Only Newman et al. (88) used the expression of self-harm without determination of definition.

Among the majority of the studies included in our systematic review, self-reported questionnaires and diagnostic interviews were used to assess the phenomenon of self-injury. The most frequently used self-reported instruments were the FASM (47, 82, 87), DSHI (80–82) and SHI (81, 86, 87). Newman et al. (88) used only one item form the NSSI-AT, and Fujimori et al. (74) applied their own developed questions. Altogether, eight different instruments were used in the 15 investigated papers, and this heterogeneity of instruments used made it difficult to compare the results of involved studies.

Related to the measurement of perfectionism, the vast majority of studies used self-reported questionnaires; only one study applied interviews (76). Although only two studies from the 15 (80, 86) measured multidimensional aspects of perfectionism with all six subscales of the FMPS, it was the most widely used questionnaire (in 7 of the 15 studies) (47, 73, 80, 81, 85–87). Other studies also measured multidimensional aspects of the FMPS but with fewer scales (73, 81, 85, 87). One-fifth [3 of the 15) of the studies measured perfectionism from self-oriented, socially prescribed, and other-oriented aspects (79, 81, 84), and a fifth (again 3 out of 15) of the studies explored only unidimensional the perfectionism (74, 75, 83). Altogether, six different instruments were used to measure this phenomenon in the 15 studies, so it made it difficult to integrate results related to this phenomenon.

Till now, relatively few studies have empirically explored the association between perfectionism and NSSI. In the 15 studies involved, we gained results both from clinical and community samples, but most studies relied on community samples, while the majority of clinical samples was enrolled from ED patients. Nock and Prinstein (79) found that among those psychiatric adolescent patients who think that other persons have high expectations of them to be perfect (SPP dimension of perfectionism), they used SMB in order to get attention, and support from others, or to avoid inconvenient situations and remove others' high expectations. Claes et al. (73) reported that women ED patients who engaged in NSSI tend to be more concerned about making mistakes, suffer from high self-criticism (ECP perfectionism, maladaptive dimension) and reported higher perceived parental criticism. Besides this, Claes et al. (73) wanted to explore the effect of adaptive perfectionism, but PSP (positive characteristics of perfectionism: setting high standards and goals) was unrelated to NSSI occurrence in women ED patients in their study. From the 15 selected relevant studies, only Nock and Prinstein (79) and Claes et al. (73) explored the function of NSSI engagement, and Claes et al. (73) show that patients with a highly self-critical and negative self-evaluation orientation (maladaptive perfectionism) tend to use NSSI as a means of self-punishment and self-torture. Regarding the association of the social function of self-injury behavior and perfectionism, Claes et al. (73) found evidence that was both consistent and inconsistent with Nock and Prinstein (79). Consistent with Nock and Prinstein (79), they found that ED patients with high maladaptive perfectionism used NSSI in order to get attention and support from other people (the cry-for-help function of NSSI). But they also found that ED patients with higher parental criticism do not use the cry-for-help function of self-injury. In this critical environment they may tend to avoid their social environment when they feel distress (73). It is inconsistent with the results of Nock and Prinstein (79), because they reported that adolescent psychiatric patients who feel that others have high expectations of them used self-injury in order to get social support and attention. Results may be influenced by the different ages of the examined study populations and by different psychiatric disorders. Nock and Prinstein (79) involved psychiatric adolescent patients with different mental disorders and with a mean age of 14.7 years, while Claes et al. (73) focused only on women ED patients with a wider age range (14–42) and with a mean age of 21.5 years. Maybe adolescent psychiatric patients are more dependent on their social environment than adult ED patients.

According to previously mentioned results, people with high maladaptive perfectionism may strive to control the situation and their environment due to the continual feelings of concern over mistakes and doubt about action (73). This attitude can be associated with the NSSI control function (interpersonal influence) (18), and perfectionistic people may use self-injury in order to get control and predictability in their environment (73). Previous results emphasize the role of social environment, parental expectation and parental perfectionism in the development of maladaptive perfectionism (39, 41, 42, 60). Claes et al. (73) also support this theory, because they reported that maladaptive perfectionism has a mediating effect between parental criticism and NSSI in female ED patients. It means that parental criticism plays an important role in the development of self-critical and negative self-evaluative orientations (maladaptive perfectionism), which can lead to NSSI engagement.

Varela-Besteiro et al. (84), Fujimori et al. (74), and Luyckx et al. (75) explored only the unidimensional aspect of perfectionism. Three research papers' results were consistent regarding maladaptive perfectionism and self-injury. Varela-Besteiro et al. (84) used adolescent ED sample, Fujimori et al. (74) adult female ED patients, and they found the same results as Claes et al. (73), who explored women ED patients. They all concluded that higher maladaptive perfectionism is significant risk factor for self-injury.

In our systematic review, only two studies (74, 75) compared how clinical and community samples relate to the association between NSSI and perfectionism. Fujimori et al. (74) and Luyckx et al. (75) used mainly ED patients in their clinical samples. Fujimori et al. (74) showed that ED patients with self-injury behavior are more perfectionistic than ED patients without self-injury and control university students. While Luyckx et al. (75) found that in both female ED and BPD patients and in female adolescent samples, there is a positive relationship between engaging in NSSI and perfectionism, but regression analysis showed a significant similar effect only in the female adolescents sample. In this study, the number of female high school students was much higher (*N* = 348) than psychiatric patients (*ED* = 80, *BPD* = 51), so the lower number of psychiatric patients in the sample might affect the power and significance of the results.

Interestingly, findings of Vieira et al. (76) do not support the relationship between perfectionism and NSSI among female ED patients. They did not find a difference between the non-NSSI group and the NSSI group related to perfectionism. Contrary to previous evidence, in which perfectionism and NSSI were assessed by self-reported questionnaires, Vieira et al. (76) measured putative risk factors for ED patients with semi-structured interviews (RFI), using questions related to self-injury and perfectionism focused on the period before the onset of eating pathology.

In community samples, the findings of Yates et al. (47) and Hoff and Muehlenkamp (80) are consistent with the results of Claes et al. (73) in women ED patients, and emphasize that maladaptive perfectionism (particularly concern over mistakes) is a potential risk factor for NSSI engagement. Moreover, critical parenting behavior may contribute to avoiding social environments (because of negative representations of others), when adolescents feel distress and this parenting behavior can increase the likelihood of NSSI (47). The study of Yates et al. (47) is the only one study in our review which measured the two phenomena both in cross-sectional and longitudinal samples. In cross-sectional sample among both girls and boys, parental critical behavior can lead to alienation toward parents, which can predict self-injurious behavior. In longitudinal sample, only among girls is it found that perceived PC in grades 6 to 8 can predict NSSI in grade 12. So, parental criticism reported by youths predicts increased self-injury over time. Interestingly, in longitudinal sample the mediation effect of parental alienation between PC and NSSI was not significant, but the longitudinal sample size was much lower than in cross-sectional sample, which can influence the power and significance of results. Hoff and Muehlenkamp (80) show that for self-injurious participants among college students are presented with difficulties in organizing and controlling over their daily lives and situations (80). Consistent with previous findings (47, 73, 79, 80), Flett et al. (81) also found higher NSSI occurrence among those female university students who perceived higher PC and felt that others have high expectations (SPP dimension of perfectionism) for them. In addition, they found among male university students that self-injury has a negative relationship with other-oriented perfectionism. Because of the different results, the authors emphasize that it would be profitable if future studies analyse and differentiate the association of self-injurious behavior in men vs. women (81). Results of Kaur and Martin (85) among medical students, Lucas et al. (86) among female college students and Chang et al. (87) among women college students also support previous evidence, which emphasizes the role of a high level of maladaptive perfectionism (especially concern over mistakes) (47, 73, 80) and high parental expectations in greater NSSI engagement (85). Chang et al. (87), similar to Claes et al. (73), found that PSP (adaptive dimension of perfectionism) has no relationship with NSSI occurrence. So, PSP plays no adaptive or protective factor in the context of NSSI behavior according to these studies. From all 15 studies, only Chang et al. (87) measured the interaction of adaptive (PSP) and maladaptive (ECP) perfectionism related to self-injurious behavior. Although the positive strivings perfectionism dimension has a positive relationship with evaluative concerns, and the PS subscale (adaptive dimension of perfectionism) was associated positively with the DA and CM subscales (maladaptive dimension of perfectionism) in their study, the interaction of PSP and ECP has no significant effect on NSSI behavior. Related to this interaction analyse authors used a 2 x 2 model of perfectionism (30) which is based on low positive striving and high evaluative concerns, so this model based on the maladaptive aspects of perfectionism, and the positive aspects of positive strivings, but previous evidence shows that the effect of positive (adaptive) perfectionism is not squarely clear, because perfectionistic strivings can have both adaptive and maladaptive consequences (27). Future research needs to analyse this interaction in more complex way accounting for NSSI behaviors, because in Chang et al. (87) study positive strivings has positive relationship with evaluative concern, and PS subscale was associated positively with DA and CM subscales, which means that people who have high standard, set higher goals, expect higher performance simultaneously feel doubt related their everyday things, and have concern over performance mistakes.

Miskey et al. (82) focused on only the cutting form of NSSI; they found that those undergraduate students who tend to ruminate (rumination scale of RI) have a longer NSSI cutting duration period. Inconsistent with previous results (73, 80), Miskey et al. (82) found that those orderly persons, who have ruminative tendencies and low concern over mistakes, have a higher NSSI cutting frequency. In this study, the rumination was the strongest predictor for NSSI frequency. Regarding these curious results, the authors suggested a deep evaluation of the content of ruminative thought as it relates to self-injurious behavior (82). In their study, maladaptive perfectionism and the need for approval of others predict significantly the age of onset of NSSI cutting. It means that the older those people are who start to engage in NSSI, the more they are concerned about mistakes and the need the approval of others (82).

Eichen et al. (83) measured only unidimensional aspects of perfectionism among college-aged women. According to their results, there is no difference regarding to perfectionism among participants with NSSI only, with NSSI/suicidal ideation, no NSSI/suicidal ideation and with suicidal ideation-only. The authors suggested that these results could be because they used the perfectionism subscale of EDI-2 (99), which does not enable a complex measurement of the dimension of perfectionism, which might show differences between investigated groups (83). Newman et al. (88) examined things from other aspects of perfectionism. According to their results, obsessive perfectionistic people have the worst mental health, with a high rate of self-injury behavior, high rumination, planfulness and compulsion tendency.

Nonsuicidal self-injury (NSSI) is a growing clinical and mental health problem, especially for youth and young adults (1–3), and both NSSI and perfectionism is a serious risk factor for suicidal ideation and behavior (28, 51, 64–68). Previous systematic and meta-analytic reviews found similar evidence related to the association between perfectionism and suicidality (suicide ideation or behavior) to what we found in our systematic review related to the relationship between NSSI and perfectionism. Maladaptive perfectionism dimensions, especially SPP, CM, DA, PC, PE are risk factors for both suicide ideation and attempts (68, 102), and meta-analytic review found that perfectionistic striving (SOP, PS) also predicts suicide ideation (102). Because there is a huge comorbidity between NSSI and suicidal behavior (28, 51, 64–68), and negative perfectionism is associated with self-injury and increased risk for suicidal behavior, our review may raise attention to appropriate treatment of perfectionistic people in order to prevent suicidal behavior when self-injurious behavior without suicidal intent has already appeared. Maladaptive perfectionism should be the focus of prevention and intervention.

Based on our systematic review we have the following suggestions for future studies in connection with the association between perfectionism and NSSI: (a) using large clinical samples with different mental disorders, (b) identifying the role of comorbidities, (c) identifying potential gender differences, (d) examining other functions of NSSI related to perfectionism, (e) monitoring the interaction between adaptive and maladaptive perfectionism dimensions, (f) using longitudinal study designs in order to assess the nature of this relationship, and (g) examining the additional plausible mechanisms behind the perfectionism-NSSI relationship.

To wrap up our main findings, our results highlight the important role of an elevated level of maladaptive perfectionism in NSSI engagement. According to studies examined, concern over mistakes and parental criticism perfectionism dimensions have the strongest effect on nonsuicidal self-injurious behavior. Our systematic review highlight that some aspects of perfectionism may influence vulnerability to NSSI.

## Limitation

Our systematic review needs to be interpreted with several limitations. From the 15 studies, only one used a longitudinal examination, while the other 14 studies had cross-sectional study designs. Therefore, no causal relationship was revealed among investigated factors. The majority of the studies included only samples of women. Only two studies investigated balanced gender ratios, so the findings may not be generalisable to both genders. Although a large body of evidence suggests that several mental disorders, both internalizing and externalizing are associated with both NSSI (6, 50–53) and also with perfectionism (24, 28, 31, 54–63), most of the 15 studies with clinical investigation enrolled ED patients; therefore, the results cannot be generalisable for all other mental disorders. Our review attempted to understand the main causes and function of NSSI engagement in connection with perfectionism through studies on clinical and non-clinical populations, but many aspects remain unclear, especially regarding the effect of the interaction of adaptive and maladaptive perfectionism related to NSSI engagement. Moreover, the heterogeneity of used instruments makes it difficult to compare the results of involved studies.

Further methodological limitation is that we involved only those papers that were written in English. Another limitation is that our systematic review is a narrative synthesis of the included relevant studies, quantitative analysis was not the objective of this study. Meta-analytic review, or meta-analysis was not performed due to the considerable heterogeneity between studies related to assessed outcomes and used measurements.

## Conclusion

To the best of our knowledge, this is the first systematic review to explore the relationship between two phenomena and clarify perfectionism as a particularly high risk factor for NSSI engagement. NSSI is a common phenomenon and is associated with elevated maladaptive perfectionism among adolescents and adults in both community and clinical samples. Furthermore, given the increased rates of NSSI and perfectionism, both are significant predictors for suicidal behavior. These phenomena are important public health issues, resulting in a growing need for a coordinated response. Perfectionistic people tend to hide behind their flawlessness mask, which makes it difficult to detect their need for help. There is an urgent need to identify effective prevention initiatives and treatment strategies aimed at these people.

## Data Availability Statement

The raw data supporting the conclusions of this article will be made available by the authors, without undue reservation.

## Author Contributions

DG: Performed the article selection process, participated in the data analyzing, presentation of the results, and discussion, drafted the manuscript. JB: Supervised the steps of the data analyzing and the selection process, drafted the manuscript. All authors read and contributed to the article and approved the final version of manuscript.

## Conflict of Interest

The authors declare that the research was conducted in the absence of any commercial or financial relationships that could be construed as a potential conflict of interest.
